# Analysis of influential factors in laparoscopic myomectomy and estimation of hidden blood loss

**DOI:** 10.3389/fsurg.2025.1614919

**Published:** 2025-10-08

**Authors:** Jingjing Lu, Lili Chu, Liliang Shen

**Affiliations:** 1 Department of Reproductive Medicine, The Affiliated People’s Hospital of Ningbo University, Ningbo, Zhejiang, China; 2 Department of Ultrasound Medicine, The Affiliated People’s Hospital of Ningbo University, Ningbo, Zhejiang, China; 3 Department of Urology, The Affiliated People’s Hospital of Ningbo University, Ningbo, Zhejiang, China

**Keywords:** laparoscopic myomectomy, influential factors, hospitalization costs, hidden blood loss, retrospective observational study

## Abstract

**Background:**

Hidden blood loss (HBL) is a critical but understudied component of perioperative blood loss in laparoscopic myomectomy, with limited data on its magnitude and associated factors. This study aimed to quantify HBL and identify its influential factors to optimize perioperative blood management and enhance patient recovery.

**Methods:**

A retrospective analysis was conducted on 139 patients who underwent laparoscopic myomectomy at The Affiliated People's Hospital of Ningbo University between January 2022 and December 2023. Demographic, surgical, and laboratory data were collected. HBL was calculated using validated formulas (Nadler, Gross, and Sehat). Univariate analyses (Kruskal–Wallis test, Pearson correlation) and multivariate linear regression were performed to identify factors associated with HBL, with statistical significance set at *p* < 0.05.

**Results:**

The mean HBL was 0.33 ± 0.02 L, accounting for 86.34% of total blood loss (TBL). Univariate analysis showed HBL was significantly associated with the number of removed fibroids (*r* = 0.172, *p* < 0.05) and their total volume (*r* = 0.202, *p* < 0.05). Multivariate regression confirmed these as independent predictors (total volume: *β* = 0.002, *p* = 0.002; number: *β* = 0.172, *p* = 0.006). Additionally, surgeon experience and senior assistants were associated with shorter operative time (*p* < 0.05), which correlated with lower hospitalization costs (*r* = 0.387, *p* < 0.001).

**Conclusion:**

HBL constitutes a substantial proportion (86.3%) of TBL in laparoscopic myomectomy, with fibroid number and total volume as key independent predictors. Optimizing surgical efficiency through experienced teams and reducing fibroid burden may help mitigate HBL and lower hospitalization costs, informing perioperative management strategies.

## Background

For millions of reproductive-aged women, uterine fibroids are more than just benign tumors—they are a source of disabling symptoms: heavy menstrual bleeding that disrupts daily life, pelvic pain that limits activity, and even infertility that shatters family plans (1). With a prevalence of over 60% by women's reproductive years (2), these growths drive a substantial need for intervention, and laparoscopic myomectomy has become the cornerstone of minimally invasive treatment. Since its first performance by Semm in 1979 (3), this approach has revolutionized care, offering shorter recovery times, fewer adhesions, and lower postoperative pain compared to open surgery (4–6)—benefits that make it the preferred choice for both patients and clinicians.

Yet, beneath these advantages lies a critical blind spot: hidden blood loss (HBL). While surgeons carefully track visible bleeding (sponges, suction), HBL—blood sequestered in tissues, hemolyzed, or trapped in the pelvic cavity—often goes unmeasured. In orthopedic surgery, this “invisible” loss is well-documented to account for 50% or more of total blood loss (TBL), leading to unexpected anemia, transfusions, and extended hospital stays (7–9). But in laparoscopic myomectomy, our understanding of HBL remains fragmented. Existing studies either mix laparoscopic and open procedures or focus on other gynecologic surgeries (10–12), leaving unanswered: How much blood is truly lost, beyond what we can see, in laparoscopic myomectomy? And which factors—fibroid size, surgeon experience, operative time—drive this hidden loss?

This knowledge gap matters. Unrecognized HBL can lead to underprepared perioperative care: patients may develop severe anemia postoperatively, requiring urgent transfusions, or face delayed recovery due to unanticipated blood loss. For clinicians, without clear data on HBL's magnitude and triggers, optimizing blood management—from preoperative planning to intraoperative hemostasis—remains guesswork.

This study aims to fill this gap in laparoscopic myomectomy. We focus exclusively on: (1) quantify HBL and its proportion of TBL; (2) identify key factors (e.g., fibroid characteristics, surgical team experience) associated with increased HBL; and (3) link these factors to clinical outcomes like operative duration and hospitalization costs. By shining a light on hidden blood loss, we seek to equip clinicians with the data needed to refine perioperative care—ultimately reducing complications and improving patient recovery.

## Methods

This retrospective observational study employed a consecutive sampling approach to enroll 139 patients who underwent laparoscopic myomectomy at The Affiliated People's Hospital of Ningbo University between January 2022 and December 2023. Consecutive sampling was adopted to minimize selection bias, ensuring all eligible patients during the study period were included unless excluded by predefined criteria. The study was approved by the institutional ethics committee (NO. 2024-057).

The inclusion criteria were as follows: (1) patients with clinically diagnosed uterine fibroids; (2) laparoscopic myomectomy was performed, and the uterine fibroid types fell under the intramural and subserosal categories; (3) a definitive diagnosis of uterine leiomyoma by postoperative pathologic examination. The exclusion criteria were as follows: (1) inadequate demographics, laboratory data, and surgical data; (2) patients with severe hematological disorders and cardiovascular disease; (3) patients with current infections or tumors; (4) uterine fibroids with particular types such as cervical myoma and broad ligament myoma; (5) with laparoscopic adenomyomectomy and laparoscopic surgery in ovarian tumors; (6) with hysteroscopic surgery.

All procedures were performed by 8 attending gynecologists (designated No. 1–No.8) with 5–12 years of specialized experience in laparoscopic gynecologic surgery. Case distribution across surgeons was as follows: No. 1 (*n* = 12, 8.6%), No. 2 (*n* = 57, 41.0%), No. 3 (*n* = 10, 7.2%), No. 4 (*n* = 8, 5.8%), No. 5 (*n* = 15, 10.8%), No. 6 (*n* = 9, 6.5%), No. 7 (*n* = 6, 4.3%), and No. 8 (*n* = 12, 8.6%). Surgeon No. 2 had the highest caseload due to her subspecialty focus on complex laparoscopic myomectomy.

Demographic data (age, height, weight, BMI, parity, cesarean section history), laboratory parameters [preoperative and postoperative day 2 hematocrit (HCT) and hemoglobin (Hb)], and surgical variables [operating surgeon, assistant, operative time, fibroid characteristics, hospitalization/surgery costs, length of stay, visible blood loss (VBL)] were extracted from electronic medical records. Fibroid volume was calculated using the ellipsoid formula:

$$\mathrm{Volume}=\frac{4\pi }{3}\times \frac{a}{2}\times \frac{b}{2}\times \frac{c}{2}=\frac{\pi \cdot a\cdot b\cdot c}{6},$$

where a, b, and c represent the three major axes measured intraoperatively with calipers.

Hidden blood loss (HBL) was calculated using validated formulas:

(1) Estimated blood volume (EBV) via Nadler's equation for females: EBV(L) = 0.3561 × height(m)^3^ + 0.03308 × weight(kg) + 0.1833; (2) Total blood loss (TBL) via Gross's formula: TBL = EBV × (HCTpre—HCTpost)/HCTave, where HCTpre is preoperative HCT (measured within 24 h preoperatively), HCTpost is postoperative day 2 HCT, and HCTave is the average of HCTpre and HCTpost; (3) HBL = TBL—VBL. Without post-operative drainage, the VBL is approximately equal to the intraoperative bleeding (13–15).

Assumptions and limitations of HBL calculation included potential HCT fluctuations due to perioperative fluid administration (≤2 L crystalloid), which may underestimate TBL, and unrecorded postoperative drainage (used selectively in 15% of cases) or delayed hemolysis beyond 48 h. For the 10 patients (7.2%) with preoperative transfusions, HCTpre was measured ≥72 h post-transfusion to avoid artifactual elevation, allowing equilibration of transfused red blood cells.

Surgical assistants were classified based on experience and proficiency: (1) Junior assistants: postgraduate year (PGY) 1–3 residents or first-year fellows with <2 years of laparoscopic myomectomy assistance experience, responsible for retraction and instrument passing under direct supervision; (2) Senior assistants: PGY 4–5 residents, senior fellows, or attending physicians with ≥2 years of experience, capable of basic laparoscopic suturing and hemostasis under indirect supervision.

Statistical analysis was performed using SPSS 25.0. Continuous variables are presented as mean ± SD (with 95% CIs) or median (IQR); categorical variables as counts (%). Univariate analyses included Kruskal–Wallis tests (comparisons across surgeons/assistants) and Pearson correlations (relationships between quantitative variables). Multivariate stepwise linear regression identified independent predictors of HBL, including variables with *p* < 0.05 and Variance Inflation Factor (VIF) around 1.0 in univariate analysis. Statistical significance was set at *p* < 0.05.

## Results

A total of 139 patients were included in this study, with demographic and surgical characteristics summarized as follows: the mean age was 41.92 ± 0.58 years (95% CI: 40.78–43.06), and the mean BMI was 23.11 ± 0.29 kg/m^2^ (95% CI: 22.54–23.68). Among the cohort, 40.3% had a history of cesarean section. The surgical sample was distributed across 8 attending gynecologists, with Surgeon No. 2 performing the largest proportion of procedures (*n* = 57, 41%), followed by Surgeon No.5 (*n* = 15, 10.8%) and others (range: 4.3%–8.6%). Most surgeries (53.2%) were conducted between 8:00–12:00, with only 4.3% starting after 17:00 (Table 1).

**Table 1:** Patient demographic and clinical information.

Parameters	Statistics
Age (year)	41.92 ± 0.58
Height (cm)	159.20 ± 4.10
Weight (kg)	58.85 ± 0.76
Body mass index (BMI) (kg/㎡)	23.11 ± 0.29
Education level	
Less than high school	71 (51.1%)
High school diploma	66 (47.5%)
College and above	2 (1.4%)
Marital status	
Single	10 (7.2%)
Married	122 (87.8%)
Divorced	6 (4.3%)
Widowed	1 (0.7%)
Gravidity	2.54 ± 0.12
Parity	1.29 ± 0.06
Length of the hospital stay	5.35 ± 0.11
History of cesarean section	
Have	83 (59.7%)
None	56 (40.3%)
Surgeon	
No. 1	4 (2.9%)
No. 2	57 (41.0%)
No. 3	14 (10.1%)
No. 4	28 (20.1%)
No. 5	6 (4.3%)
No. 6	6 (4.3%)
No. 7	9 (6.5%)
No. 8	15 (10.8%)
Assistant	
Junior assistant	57 (41.2%)
Senior assistant	82 (58.8%)
Surgery start time	
8:00 to 12:00	74 (53.2%)
12:00 to 17:00	59 (42.5%)
After 17:00	6 (4.3%)
Operative time (min)	66.87 ± 1.94
Surgery costs (Chinese yuan)	4540.29 ± 60.65
Hospitalization costs (Chinese yuan)	12343.54 ± 135.59
Preoperative Hb (g/L)	124.67 ± 1.44
Postoperative Hb (g/L)	112.91 ± 1.48
Hb loss (g/L)	11.76 ± 0.66
Preoperative HCT (L/L)	0.39 ± 0.003
Postoperative HCT (L/L)	0.35 ± 0.004
VBL (L)	0.037 ± 0.005
EBV (L)	3.58 ± 0.03
TBL (L)	0.37 ± 0.02
HBL (L)	0.33 ± 0.02
HBL/TBL (%)	86.34 ± 1.58
Number of removed fibroids	1.82 ± 0.11
Volume of the largest removed fibroid (cm^3^)	135.65 ± 9.88
Total volume of removed fibroids (cm^3^)	145.95 ± 10.21

Data are presented as *n* (%) or mean ± standard deviation. EBV, estimated blood volume; TBL, total blood loss; VBL, visible blood loss; HBL, hidden blood loss; HCT, hematocrit; Hb, hemoglobin.

Regarding fibroid characteristics, the mean number of removed fibroids was 1.82 ± 0.11 (range: 1–7), with the largest fibroid having a mean volume of 135.65 ± 9.88 cm^3^ (95% CI: 116.11–155.19) and a total volume of removed fibroids of 145.95 ± 10.21 cm^3^ (95% CI: 125.76–166.14). Operative time averaged 66.87 ± 1.94 min (95% CI: 63.05–70.69), and the mean length of hospital stay was 5.35 ± 0.11 days (95% CI: 5.14–5.57) (Table 1).

In terms of blood loss outcomes, the mean preoperative hemoglobin (Hb) was 124.67 ± 1.44 g/L (95% CI: 121.83–127.51), which decreased to 112.9 ± 1.48 g/L (95% CI: 110.0–115.8) postoperatively, resulting in a perioperative Hb loss of 11.76 ± 0.66 g/L (95% CI: 10.46–13.06). Preoperative anemia (Hb < 120 g/L) was observed in 29.5% of patients (*n* = 41), and this proportion increased to 59.7% (*n* = 83) postoperatively. The mean visible blood loss (VBL) was 0.037 ± 0.005 L (95% CI: 0.027–0.047), while the mean hidden blood loss (HBL) was 0.33 ± 0.02 L (95% CI: 0.29–0.37), accounting for 86.3% of total blood loss (TBL; 95% CI: 83.2–89.5%).

**Figure 1:**
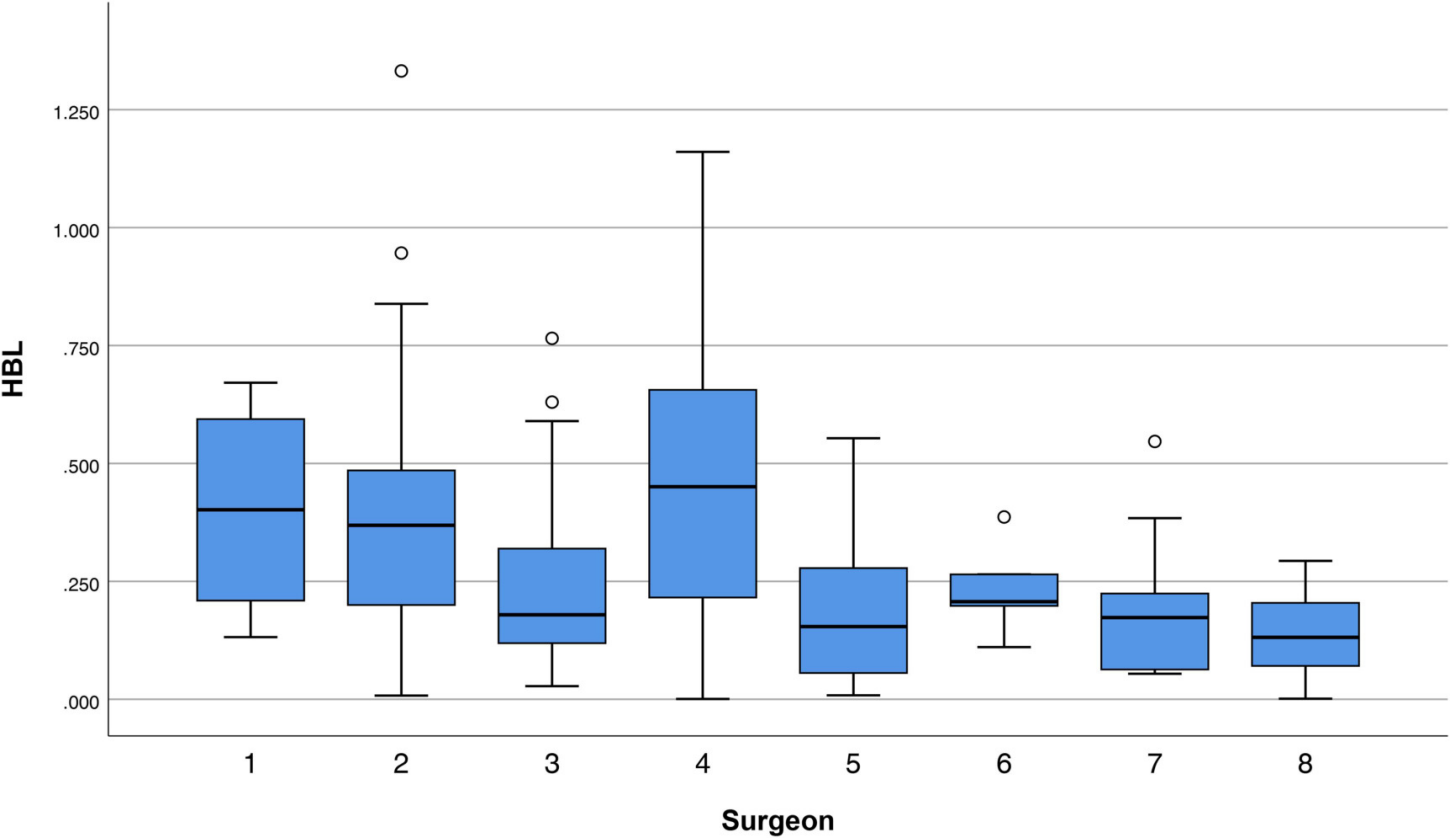
A boxplot illustrating HBL variability across surgeons.

**Figure 2:**
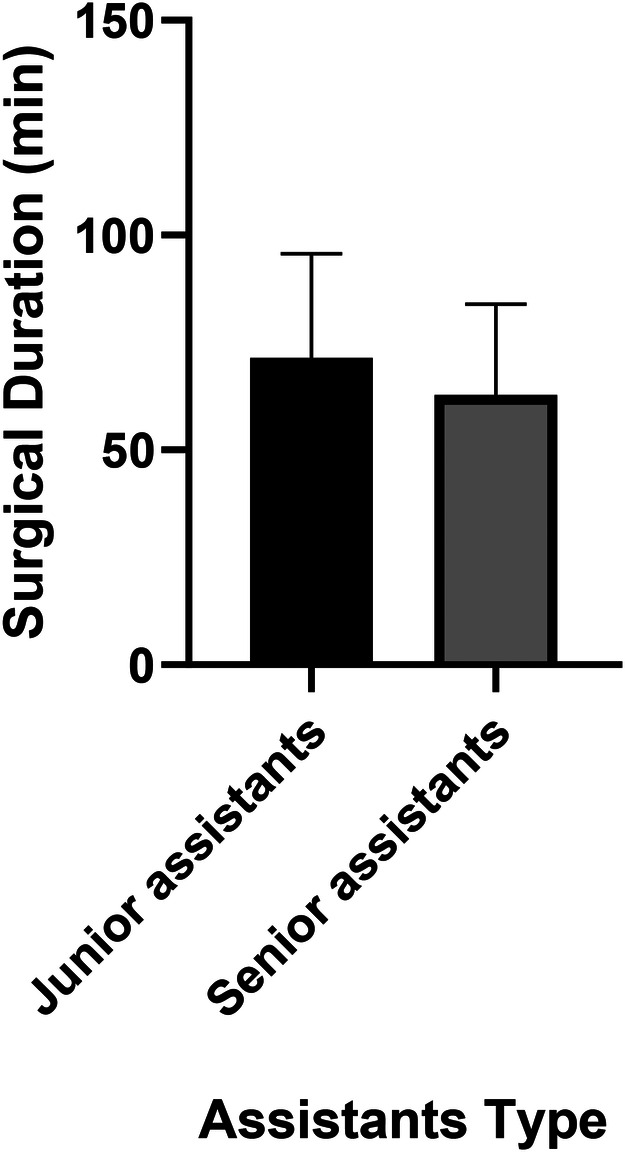
A bar chart comparing operative time between senior and junior assistants. Senior assistants were associated with shorter operative times than junior assistants (63.1. ± 21.4 vs. 71.8 ± 23.8 min; *p* < 0.05).

Univariate analyses revealed significant variability among surgeons in several outcomes: perioperative Hb loss (*p* < 0.001), operative duration (*p* = 0.028), length of stay (*p* = 0.007), VBL (*p* < 0.001), HBL (*p* = 0.001), and TBL (*p* = 0.001) (Table 2). For example, Surgeon No. 2 had the shortest operative time (60.5 ± 19.5 min), while Surgeon No. 8 had the longest (81.0 ± 24.2 min) but the lowest HBL (0.14 ± 0.10l), with a moderate effect size compared to Surgeon No.4 (0.46 ± 0.29 L) (Figure 1; Table 2). Senior assistants were associated with shorter operative times than junior assistants (63.1. ± 21.4 vs. 71.8 ± 23.8 min; *p* < 0.05) (Figure 2) but showed no significant correlation with HBL (*p* = 0.929) (Table 3). Surgery start time had no impact on any outcome (*p* > 0.05) (Table 4).

**Table 2:** The effect of the different surgeons on the surgery.

Variable	*P* Value
Hb loss	<0.001
Hospitalization costs	0.204
Surgery costs	0.485
Operative time	0.028
Length of stay	0.007
VBL	<0.001
HBL	0.001
TBL	0.001

**Table 3:** Effect of different assistants on surgery.

Variable	*P* Value
Hb loss	0.445
Hospitalization costs	0.318
Surgery costs	0.749
Surgery duration	0.041
Length of stay	0.516
VBL	0.347
HBL	0.929
TBL	0.865

**Table 4:** Effect of start time of surgery on surgery.

Variable	*P* Value
Hb loss	0.826
Hospitalization costs	0.201
Surgery costs	0.258
Surgery duration	0.330
Length of the hospital stay	0.553
VBL	0.335
HBL	0.553
TBL	0.590

Pearson correlation analysis demonstrated that HBL was positively associated with the number of removed fibroids (*r* = 0.172, *p* < 0.05) and total fibroid volume (*r* = 0.202, *p* < 0.05; Table 5). Multivariate regression further confirmed total fibroid volume (*β* = 0.002, *p* = 0.002) and the number of fibroids (*β* = 0.172, *p* = 0.006) as independent predictors of log-transformed HBL (Table 6).

**Table 5:** Pearson correlation analysis of quantitative data.

Paired quantitative data name	Age	Height	Weight	BMI	Hospitalization costs	Surgery costs	Operative time	Length of stay	Number of removed fibroids	Volume of the largest removed fibroids	Total volune of removed fibroids	VBL	HBL	TBL	Hb loss
Age	1														
Height	−0.159	1													
Weight	−0.095	.273**	1												
BMI	−0.03	−0.137	.914**	1											
Hospitalization costs	0.041	0.158	0.164	0.1	1										
Surgery costs	0.029	0.122	0.096	0.049	.637**	1									
Operative time	−0.058	0.052	0.127	0.099	.387**	.221**	1								
Length of stay	0.038	0.049	0.101	0.086	.542**	.191*	.334**	1							
Number of removed fibroids	.171*	−0.107	−0.033	0.01	−0.003	0.111	.201*	−0.094	1					.	
Volume of the largest removed fibroids	−0.028	.182*	0.076	0.005	.254**	0.063	.251**	0.042	−0.081	1					
Total volune of removed fibroids	−0.011	.180*	0.079	0.008	.259**	0.08	.310**	0.021	0.079	.965**	1				
VBL	0.055	−0.025	0.04	0.053	.269**	0.131	.459**	0.103	.326**	0.125	.241**	1			
HBL	0.078	−0.025	−0.142	−0.134	0.086	−0.009	0.113	−0.018	.172*	0.137	.202*	.229**	1		
TBL	0.085	−0.029	−0.121	−0.111	0.143	0.022	.212*	0.008	.236**	0.156	.243**	.446**	.973**	1	
Hb loss	0.07	−0.086	−.205*	−.181*	0.066	0.005	.232**	−0.063	.232**	0.165	.245**	.369**	.895**	.910**	1

* Signify that the correlation is significant at the 0.05 level (2-tailed).

** Signify that the correlation is significant at the 0.01 level (2-tailed).

**Table 6:** Multiple regression model for log-transformed HBL.

Predictor	Coefficient (B)	Standard error	Standardized coefficient (β)	*t*-value	*P*-value	Tolerance	VIF
Intercept (Constant)	−4.633	0.165		−27.995	<0.001		
Total volune of removed fibroids	0.002	0.001	0.262	3.237	0.002	0.994	1.006
Number of removed fibroids	0.172	0.062	0.225	2.776	0.006	0.994	1.006

*R*^2^ = 0.128; Adjusted *R*^2^ = 0.115; Standard error of the estimate = 0.111; ANOVA: F = 123.897; VIF, variance inflation factor.

Hospitalization costs averaged 12343.54 ± 135.59 yuan (95% CI: 12077.0–12610.1) and correlated significantly with operative time (*r* = 0.387, *p* < 0.01), length of stay (*r* = 0.542, *p* < 0.01), and total fibroid volume (*r* = 0.259, *p* < 0.01).

## Discussion

This study highlights that hidden blood loss (HBL) constitutes 86.3% of total blood loss (TBL) in laparoscopic myomectomy, emphasizing its critical role in perioperative blood management. This proportion is notably higher than the 71.5% reported by Ye et al. in a mixed cohort of laparotomic and laparoscopic myomectomy (16), a discrepancy likely explained by methodological differences: Ye et al. included postoperative drainage volume in visible blood loss (VBL), whereas our study excluded inconsistently recorded drainage, potentially underestimating VBL and inflating HBL. Additionally, our cohort exclusively underwent laparoscopic surgery, and the confined pelvic space in laparoscopy may trap more blood in tissues or cavities, increasing HBL relative to visible losses—consistent with Masakazu Sato et al.'s observation that laparoscopic bleeding is prone to underestimation (17).

Sehat et al.'s foundational work on HBL in orthopedics (18) established that invisible loss drives clinical outcomes, and our findings extend this to gynecologic surgery: HBL independently correlated with fibroid number and total volume, with larger or more numerous fibroids associated with greater HBL. This aligns with Ye et al.'s identification of leiomyoma number as a risk factor (16) but adds specificity by isolating laparoscopic cases and confirming total volume as a key predictor—likely due to increased vascularity and dissection time in larger fibroids (19). This has practical implications for preoperative counseling: patients with multiple or large fibroids may benefit from tailored blood management strategies, such as preoperative iron supplementation, erythropoietin usage or proactive transfusion planning. However, randomized controlled trial (RCT) evidence indicates intravenous administration of tranexamic acid in patients undergoing laparoscopic or robotic myomectomies was not associated with decreased blood loss (20).

Surgeon variability in HBL and operative time underscores the role of experience: Surgeon No.8, despite longer operative times, had lower HBL, potentially reflecting superior hemostatic suturing techniques. Similarly, senior assistants reduced operative time by 8.73 min, a finding with cost implications given the correlation between operative time and hospitalization expenses (*r* = 0.387, *p* < 0.01). However, operative time didn't correlate with HBL in univariate analysis and not as an independent predictor, suggesting its possible influence is mediated by fibroid burden. This contrasts with Wen et al.'s report that time directly drives HBL in cervical and lumbar surgery (15, 21), likely due to differences in tissue vascularity between gynecologic and spinal procedures.

While our study focuses on HBL, it is critical to acknowledge the potential overlap between uterine fibroids and malignant lesions. Contemporary research underscores that leiomyosarcoma (LMS) represents the most prevalent sarcoma arising within the myometrium (22). This malignancy is susceptible to misdiagnosis as uterine leiomyomas, necessitating computed tomography (CT) imaging of the thoracic, abdominal, and pelvic regions, while surgical re-exploration should also be entertained as a clinical consideration (23). Although none of our patients had postoperative sarcoma diagnoses, this risk underscores the importance of preoperative imaging (e.g., MRI to assess tumor vascularity) and intraoperative frozen section analysis for suspicious lesions (24, 25). Surgeons must balance thorough hemostasis with vigilance for atypical findings, as delayed sarcoma detection could compromise oncologic outcomes.

In recent decades, laparoscopic surgery has emerged as a prominent alternative to the conventional laparotomic approach for myomectomy. A latest meta-analysis demonstrates that laparoscopic myomectomy confers several advantages, such as shorter hospital stays, reduced intraoperative blood loss, and decreased postoperative analgesic consumption, when compared with the laparotomic approach. However, no statistically significant differences were observed in the rates of intraoperative or postoperative complications between the two approaches. Additionally, there were no statistically significant disparities in pregnancy rates or other obstetric outcomes (26).

Limitations include the single-center retrospective design, inconsistent recording of postoperative drainage (potentially biasing HBL calculations), and lack of stratification by fibroid subtype (intramural vs. subserosal). Future multi-center prospective studies should address these to validate findings and explore HBL mechanisms in gynecologic surgery.

## Conclusion

In laparoscopic myomectomy, hidden blood loss constitutes 86.3% of total blood loss, with fibroid number and total volume as independent predictors. Surgeon and assistant experience reduces operative time and hospitalization costs but does not directly affect HBL. These findings inform perioperative blood management and cost optimization via surgical team training and preoperative fibroid volume reduction.

## Data Availability

The raw data supporting the conclusions of this article will be made available by the authors, without undue reservation.
